# Burden of Ischaemic heart disease and attributable risk factors in China from 1990 to 2015: findings from the global burden of disease 2015 study

**DOI:** 10.1186/s12872-018-0761-0

**Published:** 2018-02-02

**Authors:** Ganshen Zhang, Chuanhua Yu, Maigeng Zhou, Lu Wang, Yunquan Zhang, Lisha Luo

**Affiliations:** 1 Department of Epidemiology and Biostatistics, School of Health Sciences, Wuhan University, #185 Donghu Road, Wuhan, Hubei 430071 China; 2 National Center for Chronic and Non-communicable Disease Control and Prevention, Beijing, China

**Keywords:** Ischaemic heart disease, Mortality, Disability-adjusted life years, Risk factors

## Abstract

**Background:**

Ischaemic heart disease (IHD) is a major barrier to sustainable human development, but its health burden and geographic distribution among provinces of China remain unclear. This study aimed to estimate IHD burden in provinces of China, and attributable to risk factors from 1990 to 2015.

**Methods:**

Data were collected from the Global Burden of Disease 2015 Study, which evaluated IHD burden and attributable risk factors using deaths and disability-adjusted life years (DALYs). Statistical models including cause of death ensemble modelling, Bayesian meta-regression analysis, and comparative risk assessment approaches were applied to reduce bias and produce comprehensive results of IHD deaths, DALYs and attributable risks. The 95% uncertainty intervals (UIs) were calculated and reported for mortality and DALYs.

**Results:**

The age-standardised death rate per 100,000 people increased by 13.3% from 101.3 (95%UI: 95.3–107.5) to 114.8 (95%UI: 109.8–120.1) from 1990 to 2015 in China, whereas the age-standardised DALY rate declined 3.9% to 1760.2 per 100,000 people (95%UI: 1671.6–1864.3). In 2015, the age-standardised death rate per 100,000 people was the highest in Heilongjiang (187.4, 95%UI: 161.6–217.5) and the lowest in Shanghai (44.2, 95%UI: 37.0–53.1), and the age-standardised DALY rate per 100,000 people was the highest in Xinjiang (3040.8, 95%UI: 2488.8–3735.4) and the lowest in Shanghai (524.4, 95%UI: 434.7–638.4). Geographically, the age-standardised death and DALY rates for southern provinces were lower than northern provinces, especially in southeastern coastal provinces. 95.3% of the IHD burden in China was attributable to environmental, behavioural and metabolic risk factors. The five leading IHD risks in 2015 were high systolic blood pressure, high total cholesterol, diet high in sodium, diet low in whole grains, and smoking.

**Conclusions:**

Population growth and ageing has led to a steady increase in the IHD burden. Regional disparities in IHD burden were observed in provinces of China. The distribution characteristics of IHD burden provide guidance for decision makers to formulate targeted preventive policies and interventions.

**Electronic supplementary material:**

The online version of this article (10.1186/s12872-018-0761-0) contains supplementary material, which is available to authorized users.

## Background

Cardiovascular diseases (CVDs), which accounted for one-third of global deaths in 2015, are recognised as a major barrier to sustainable human development [1, 2]. Of all CVDs, ischaemic heart disease (IHD) is one of the leading causes of mortality and disease burden worldwide [3], resulting in approximately 8.9 million deaths and 164.0 million disability-adjusted life years (DALYs) globally in 2015 [4–6]. A number of risk factors have been found to be associated with IHD such as hypertension, high total cholesterol, diet high in sodium, and smoking [7–9]. Additionally, mortality from IHD has decreased in developed countries in the past three decades [10] but has increased continuously in many low- and middle- income countries [11–14].

China, one of the largest developing countries in the world, has undergone rapid health transition in the past decades under the background of economic growth and social change. The prevalence and mortality of non-communicable diseases (NCDs), including IHD, has been rising rapidly in China [15]. Therefore, the Chinese Government has implemented the Plan of Healthy China 2030 in response to the Sustainable Development Goals (target 3.4.1) by the United Nations to reduce premature mortality from major NCDs [16, 17]. Furthermore, IHD is one of the major health threats to Chinese people and was the second leading causes of mortality next to stroke in 2013 [18]. However, IHD burden and its geographic distribution among provinces of China remain unclear due to the lack of comparable data. The Global Burden of Diseases, Injuries, and Risk Factors 2015 Study (GBD 2015) estimated health lost from fatal and non-fatal outcomes by integrating all available data on incidence, prevalence, and mortality to produce accurate, consistent, transparent, and up-to-date estimates for global, regions, nations, and subnational regions for some countries [2, 19]. In this study, we aimed to estimate the disease burden of IHD on mortality and DALYs in various provinces of China, as well as DALYs attributed to IHD risk factors, by using the data from the GBD 2015 study.

## Methods

### Data sources

Data for this study were obtained from the GBD 2015 study, which estimated the burden of 315 diseases and injuries, 2619 unique sequelae, and 79 risk factors in 195 countries worldwide from 1990 to 2015 [4–7]. Original data from three main data sources including the Disease Surveillance Points (DSPs), the Maternal and Child Surveillance System, and the Chinese Center for Disease Control and Prevention (CDC) Cause of Death Reporting System were adapted by the GBD 2015 collaborators to estimate outcomes of IHD in China. IHD cases were identified using the World Health Organization clinical criteria and the 10th revision of the International Classification of Diseases and Injuries (ICD-10) discharge diagnosis codes (codes I20-I25).

### Mortality, disability-adjusted life years and risk factors

The general methodological approaches of GBD 2015 and the specific methodology used to study IHD in China have been described elsewhere [4–7]. We used mortality and DALYs to measure the burden caused by IHD. DALYs from IHD consisted of two parts, years of life lost (YLLs) that quantifies life loss caused by premature mortality from fatal IHD and years lived with disability (YLDs) that evaluates health loss from living with non-fatal IHD sequelae such as non-fatal myocardial infarction, angina, and ischaemic heart failure [6, 20]. Cause of Death Ensemble modelling (CODEm) was the principal method adapted to estimate fatal IHD mortality and YLLs [4]. Prevalence for non-fatal IHD sequelae was estimated using Bayesian meta-regression analysis using the DisMod-MR 2.1 software, and its disability weight was evaluated by population-based health surveys and an open web-based survey [5, 21]. We computed DALYs via the summation of YLLs and YLDs [5]. Comparative risk assessment (CRA) approaches were used to evaluate the number of excess DALYs from IHD observed in a given year that can be attributed to past exposure to a risk factor [7]. We used the revised GBD 2015 global population age standard to determine all the age-standardised death and DALY rates.

### Uncertainty interval

For each estimated rate and number of deaths and DALYs, we reported its 95% uncertainty interval (UI), which was estimated by taking 1000 samples from the posterior distribution of each quantity and using the 25th and 975th-ordered draws of the uncertainty distribution [4–7].

## Results

### Mortality

The IHD death rate per 100,000 people in China increased significantly from 52.2 (95%UI: 49.2–55.6) in 1990 to 105.6 (95%UI: 101.0–110.8) in 2015. A total of 1461.2 thousand IHD deaths (95%UI: 1396.8–1532.9) occurred in 2015 in China compared to 6,03.1 thousand in 1990. IHD deaths accounted for 15.5% of all deaths in 2015, ranking only next to stroke (20.1%), with a sharp increase from 6.8% in 1990. Nevertheless, the age-standardised death rate only increased slightly in both males (19.5%) and females (4.5%) from 1990 to 2015. Of all IHD deaths in 2015, 58.0% were in males and 66.4% were in those aged 70+ years.

**Fig. 1:**
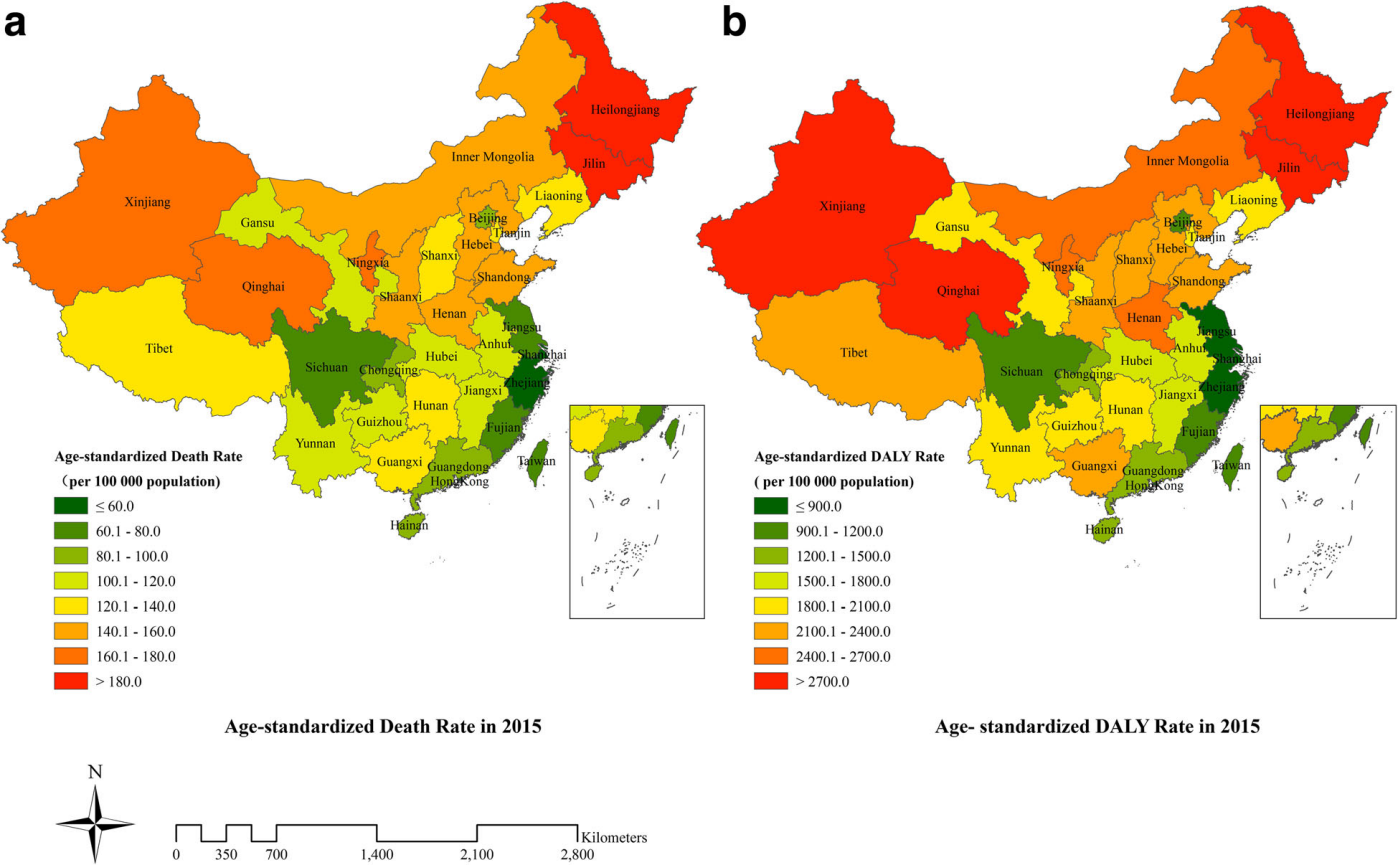
Age-standardised death rate (**a**) and age-standardised DALY rate (**b**) from ischaemic heart disease for both sexes combined in 2015 in China. DALYs = disability-adjusted life years

Table 1 provides the number of deaths and age-standardised death rates from IHD in various provinces of China from 1990 to 2015. In 2015, Shandong had the greatest number of IHD deaths at 146.4 thousand (95%UI: 123.6–171.5), and Heilongjiang had the highest age-standardised death rate at 187.4 per 100,000 people (95%UI: 161.6–217.5) followed by Jilin and Xinjiang. The lowest age-standardised death rate occurred in Shanghai with 44.2 per 100,000 people (95%UI: 37.0–53.1) followed by Zhejiang and Hong Kong. Age-standardised death rates for southern provinces were lower than northern provinces, especially in southeastern coastal provinces such as Shanghai, Zhejiang, Hong Kong, Jiangsu, and Fujian (Fig. 1a). Age-standardised death rates for 22 provinces increased while the remaining 11 provinces decreased between 1990 and 2015; the highest increases occurred in Qinghai (54.0%), Shandong, and Hunan, and the largest declines were observed in Macao (− 52.3%), Hong Kong, and Beijing.

**Table 1:** Number of deaths and age-standardised death rates from ischaemic heart disease with percent change, 1990–2015, in provinces of China

Locations	Number of deaths (thousand)			Age-standardized death rate per 100,000 people		
	1990	2015	% change	1990	2015	% change
China	603.1 (568.0–642.2)	1461.2 (1396.8–1532.9)	142.3	101.3 (95.3–107.5)	114.8 (109.8–120.1)	13.3
Anhui	22.8 (18.4–28.7)	65.2 (55.3–76.8)	186.0	86.6 (69.9–106.1)	107.8 (91.7–126.1)	24.4
Beijing	7.5 (6.0–9.2)	16.9 (14.6–19.7)	125.7	116.7 (94.9–141.2)	86.5 (74.7–100.6)	−25.9
Chongqing	6.5 (5.2–8.1)	28.3 (22.0–34.7)	332.3	73.2 (58.9–89.8)	87.3 (69.1–105.8)	19.3
Fujian	11.0 (8.9–13.6)	22.7 (18.5–27.1)	105.9	78.0 (64.0–94.8)	70.7 (57.7–84.1)	−9.4
Gansu	7.8 (6.2–9.6)	23.3 (19.0–27.9)	197.0	87.8 (71.3–105.5)	117.3 (97.6–138.2)	33.6
Guangdong	30.4 (24.1–37.5)	79.7 (69.2–92.2)	162.3	94.9 (75.8–116.1)	97.7 (85.0–112.7)	2.9
Guangxi	21.7 (17.5–26.9)	61.1 (50.6–72.8)	181.6	98.6 (79.4–121.7)	136.3 (113.4–161.7)	38.3
Guizhou	12.7 (10.1–16.0)	34.5 (27.1–43.7)	172.1	87.7 (70.3–109.8)	114.6 (91.0–143.0)	30.7
Hainan	4.0 (3.2–4.9)	7.5 (6.0–9.3)	88.2	120.2 (98.6–145.4)	91.5 (72.7–113.2)	−23.9
Hebei	38.4 (30.0–48.5)	92.7 (77.1–111.7)	141.6	114.3 (90.3–141.6)	143.5 (120.8–169.7)	25.5
Heilongjiang	28.2 (21.9–35.3)	66.7 (56.3–78.7)	136.4	198.0 (158.3–242.3)	187.4 (161.6–217.5)	−5.4
Henan	55.1 (44.0–67.2)	121.7 (101.2–146.6)	120.8	119.8 (96.2–144.7)	152.4 (127.7–181.4)	27.2
Hong Kong Special Administrative Region of China	4.2 (3.9–4.7)	7.4 (6.6–8.7)	78.6	91.7 (85.5–103.6)	58.3 (52.1–67.7)	−36.4
Hubei	27.5 (22.1–33.9)	58.4 (49.1–69.1)	112.5	106.5 (86.8–129.6)	111.4 (94.8–131.3)	4.6
Hunan	31.1 (24.6–38.6)	93.5 (79.5–110.2)	201.0	99.4 (79.8–121.7)	139.2 (118.1–163.3)	40.0
Inner Mongolia	13.7 (10.8–17.4)	32.3 (26.7–38.5)	136.5	157.0 (127.8–193.9)	158.7 (134.1–184.1)	1.1
Jiangsu	23.5 (18.5–29.7)	55.7 (47.1–65.2)	137.0	59.5 (47.3–74.7)	63.4 (53.8–74.2)	6.5
Jiangxi	17.1 (13.6–21.3)	38.2 (31.8–45.3)	123.4	101.6 (81.5–124.5)	106.4 (89.3–124.9)	4.8
Jilin	22.3 (17.7–28.0)	45.4 (36.5–56.8)	103.3	201.9 (165.0–248.9)	182.0 (148.4–223.1)	−9.9
Liaoning	28.1 (22.3–34.6)	66.0 (56.7–77.2)	134.8	129.6 (104.3–156.8)	135.7 (117.6–157.1)	4.7
Macao Special Administrative Region of China	0.41 (0.36–0.46)	0.43 (0.34–0.54)	6.0	174.0 (153.2–194.4)	83.1 (64.7–103.2)	−52.3
Ningxia	2.0 (1.5–2.4)	7.2 (5.6–9.1)	266.0	122.0 (97.7–148.7)	166.8 (133.8–206.2)	36.7
Qinghai	1.8 (1.4–2.2)	6.6 (5.1–8.5)	278.8	114.6 (91.5–141.6)	176.5 (140.2–218.6)	54.0
Shaanxi	17.1 (13.7–21.2)	47.3 (36.0–58.8)	177.5	113.0 (91.8–138.0)	151.9 (119.6–184.4)	34.5
Shandong	50.3 (41.0–61.2)	146.4 (123.6–171.5)	191.0	103.8 (84.0–125.1)	145.9 (123.6–170.4)	40.6
Shanghai	5.9 (4.7–7.1)	13.1 (11.0–15.7)	123.2	57.6 (46.3–69.2)	44.2 (37.0–53.1)	−23.3
Shanxi	16.6 (13.2–20.8)	41.0 (31.4–51.5)	146.5	114.9 (92.9–141.9)	137.4 (108.5–168.8)	19.6
Sichuan	47.7 (38.6–59.5)	65.4 (51.9–80.8)	37.0	88.1 (72.2–108.4)	75.4 (60.3–92.4)	−14.4
Tianjin	6.8 (5.5–8.5)	16.8 (14.2–19.6)	145.5	126.4 (103.3–153.2)	134.8 (115.6–155.4)	6.7
Tibet	1.4 (1.1–1.9)	2.3 (1.8–2.8)	61.0	138.6 (107.7–185.2)	135.6 (110.3–168.7)	−2.1
Xinjiang	10.9 (8.6–13.8)	27.1 (22.2–33.2)	148.0	169.5 (137.3–211.1)	178.4 (148.5–214.2)	5.2
Yunnan	14.7 (11.7–18.5)	44.6 (37.1–54.3)	202.9	86.0 (68.6–107.2)	118.2 (99.2–142.2)	37.4
Zhejiang	14.0 (11.3–17.1)	26.0 (21.3–31.2)	85.6	57.1 (46.5–68.9)	45.0 (37.0–53.8)	−21.2

### YLLs and YLDs

In 2015, YLLs from IHD was 24,407.2 thousand person years (95%UI: 23,041.1–25,809.1), which increased by 84.6% from 13,221.8 thousand (95%UI: 12,382.0–14,098.7) in 1990. However, the age-standardised YLL rate per 100,000 people decreased 4.3% from 1761.1 (95%UI: 1656.4–1877.2) in 1990 to 1671.9 (95%UI: 1585.1–1763.4) in 2015 (Table 2). Among all provinces, Xinjiang had the highest age-standardised YLL rate at 2956.7 (95%UI: 2403.7–3659.6) in 2015, and the lowest occurred in Shanghai at 427.6 (95%UI: 346.8–536.0). Age-standardised YLL rates for 20 provinces decreased whereas the remaining 13 provinces increased from 1990 to 2015; Macao showed the most rapid decline while Qinghai experienced the most rapid increase.

**Table 2:** Age-standardised YLL and YLD rates and YLLs/YLDs ratios for ischaemic heart disease in 1990 and 2015 in provinces of China

Province	Age-standardized YLL rate per 100,000 people			Age-standardized YLD rate per 100,000 people			YLL/YLD ratio 1990	YLL/YLD ratio 2015
	1990	2015	% change	1990	2015	% change		
China	1761.1 (1656.4–1877.2)	1671.9 (1585.1–1763.4)	−4.3	71.3 (47.9–97.7)	88.3 (59.4–120.9)	23.8	25.2	18.0
Anhui	1426.7 (1137.0–1795.5)	1505.4 (1249.9–1812.1)	5.5	70.9 (48.1–97.7)	87.5 (58.4–120.7)	23.5	19.8	16.4
Beijing	1849.3 (1477.0–2306.9)	1040.6 (873.1–1251.1)	−43.7	90.4 (61.4–124.2)	111.3 (76.0–153.9)	23.0	20.3	8.9
Chongqing	1216.8 (968.3–1527.6)	1215.7 (925.1–1533.8)	−0.1	63.7 (42.3–88.3)	79.2 (52.9–110.1)	24.4	18.7	14.5
Fujian	1307.5 (1054.8–1636.3)	925.6 (738.6–1128.8)	− 29.2	68.6 (46.4–94.4)	84.2 (56.7–115.8)	22.8	19.0	10.3
Gansu	1565.7 (1239.1–1908.2)	1755.2 (1422.3–2120.7)	12.1	64.7 (43.4–88.8)	80.4 (54.3–110.5)	24.3	24.6	20.3
Guangdong	1611.02 (1259.93–2005.35)	1272.8 (1087.1–1502.2)	−21.0	74.2 (50.4–102.4)	91.3 (61.7–125.6)	23.2	22.1	13.2
Guangxi	1719.6 (1377.1–2179.6)	2152.1 (1748.0–2640.7)	25.2	68.2 (46.0–94.0)	83.9 (56.8–115.8)	23.1	25.5	25.1
Guizhou	1510.6 (1197.1–1917.9)	1845.2 (1434.4–2379.7)	22.2	61.7 (41.3–85.7)	76.9 (51.3–106.5)	24.6	24.2	23.0
Hainan	1957.9 (1559.9–2444.0)	1315.4 (1006.4–1708.9)	−32.8	67.3 (45.2–93.1)	83.1 (56.2–114.9)	23.4	28.6	15.5
Hebei	2065.0 (1598.0–2632.7)	2255.2 (1846.2–2780.1)	9.2	73.2 (49.8–100.8)	90.0 (60.4–124.5)	22.9	29.4	23.9
Heilongjiang	3606.6 (2791.8–4496.9)	2761.8 (2291.2–3302.4)	−23.4	80.2 (54.6–109.3)	98.2 (67.1–133.4)	22.4	49.7	26.1
Henan	2173.8 (1734.0–2676.7)	2324.5 (1903.3–2855.6)	6.9	75.6 (50.8–104.0)	93.1 (63.6–127.8)	23.1	30.2	24.0
Hong Kong Special Administrative Region of China	1298.6 (1228.1–1434.1)	699.7 (624.8–794.3)	−46.1	71.2 (48.1–99.1)	86.3 (57.2–119.3)	21.2	17.7	8.5
Hubei	1840.91 (1471.74–2290.86)	1577.4 (1308.6–1890.7)	−14.3	68.7 (46.3–94.5)	84.6 (57.0–116.4)	23.1	26.9	17.1
Hunan	1733.0 (1364.6–2157.8)	2004.2 (1664.3–2402.9)	15.7	65.3 (43.5–89.9)	80.2 (53.7–112.1)	22.9	26.6	23.8
Inner Mongolia	2829.3 (2252.6–3587.8)	2371.5 (1935.4–2863.1)	−16.2	78.7 (53.5–108.4)	96.3 (64.9–132.2)	22.2	38.6	23.3
Jiangsu	928.2 (724.2–1179.2)	787.0 (653.1–944.7)	− 15.2	71.8 (47.8–98.3)	88.3 (59.7–121.3)	23.0	12.6	8.3
Jiangxi	1703.8 (1351.9–2135.7)	1482.1 (1206.5–1773.0)	−13.0	67.5 (45.4–92.5)	82.8 (55.9–113.3)	22.8	25.0	16.5
Jilin	3586.3 (2839.1–4495.1)	2654.5 (2088.9–3408.8)	− 26.0	80.4 (54.4–110.8)	97.9 (67.1–135.1)	21.8	47.4	25.0
Liaoning	2236.8 (1763.7–2773.4)	1875.8 (1578.9–2236.1)	−16.1	84.9 (57.9–115.7)	103.8 (70.2–142.9)	22.2	27.6	16.7
Macao Special Administrative Region of China	2589.3 (2267.0–2883.6)	988.1 (763.2–1240.8)	−61.8	90.3 (61.2–124.2)	110.1 (74.7–151.8)	21.9	28.6	8.2
Ningxia	2085.0 (1626.4–2558.8)	2552.9 (1963.4–3248.2)	22.4	78.5 (53.0–108.3)	96.5 (65.4–132.3)	22.9	27.7	25.3
Qinghai	2092.9 (1642.2–2671.4)	2793.5 (2127.3–3591.7)	33.5	63.3 (42.0–87.5)	79.0 (53.2–109.8)	24.8	34.6	33.3
Shaanxi	1993.6 (1603.5–2489.0)	2216.3 (1659.0–2795.6)	11.2	72.2 (49.0–99.3)	89.8 (60.3–124.4)	24.3	28.6	22.8
Shandong	1808.6 (1466.0–2224.1)	2043.7 (1698.1–2462.8)	13.0	77.0 (52.2–106.2)	95.0 (64.8–130.1)	23.4	24.1	20.3
Shanghai	802.7 (640.8–982.9)	427.6 (346.8–536.0)	−46.7	78.5 (53.2–108.0)	96.8 (65.7–133.2)	23.3	9.7	4.1
Shanxi	2092.7 (1651.9–2621.9)	2117.0 (1592.1–2701.9)	1.2	75.3 (51.1–104.3)	92.8 (63.2–126.9)	23.2	29.2	21.9
Sichuan	1468.84 (1184.9–1844.6)	1092.6 (841.8–1371.1)	− 25.6	60.6 (40.1–83.0)	75.0 (49.9–103.5)	23.7	23.4	13.7
Tianjin	2205.8 (1759.3–2747.8)	1780.0 (1492.3–2154.6)	−18.4	90.8 (62.2–125.1)	112.2 (77.7–153.3)	23.6	25.5	15.1
Tibet	2682.3 (2055.1–3581.9)	2257.6 (1809.1–2847.6)	−15.8	57.0 (38.1–79.9)	71.2 (47.2–98.9)	24.9	49.3	31.8
Xinjiang	3177.8 (2481.1–4037.5)	2956.7 (2403.7–3659.6)	− 7.0	68.0 (45.9–93.0)	84.0 (56.0–115.7)	23.5	50.0	35.0
Yunnan	1526.2 (1205.6–1927.2)	1884.4 (1537.7–2325.8)	23.5	63.2 (42.4–87.7)	78.3 (52.4–109.8)	23.8	24.3	23.4
Zhejiang	864.4 (685.2–1069.9)	552.3 (446.4–673.8)	− 36.1	68.4 (45.9–94.7)	83.7 (55.8–114.3)	22.4	12.1	6.2

YLLs = years of life lost, YLDs = years lived with disability

IHD caused an estimated 1358.0 thousand YLDs (95%UI: 915.7–1870.3) in China in 2015, which was 2.5 times higher than the YLDs in 1990 at 524.6 thousand (95%UI: 352.5–720.2). The age-standardised YLD rate per 100,000 people rose 23.8% from 71.3 (95%UI: 47.9–97.7) to 88.3 (95%UI: 59.4–120.9) (Table 2). All provinces experienced increases in age-standardised YLD rates between 1990 and 2015. In 2015, the highest age-standardised YLD rate appeared in Tianjin, followed by Beijing and Macao, whereas the lowest YLD rate occurred in Tibet.

For IHD, YLLs was much higher than YLDs, as the YLLs/YLDs ratio for IHD was 25.2 in 1990 and reduced to 18.0 in 2015. Although the ratios decreased for all provinces during 1990 to 2015, they remained greater than 10 for most provinces.

### DALYs

IHD caused a total of 2576.5 thousand DALYs (95%UI: 2439.1–2737.4) in China in 2015, accounting for 7.5% of total DALYs from all causes, ranking only second to stroke (10.1%). The IHD DALY rate per 100,000 people increased from 1190.7 (95%UI: 1120.9–1268.9) in 1990 to 1862.4 (95%UI: 1763.1–1978.6) in 2015. However, the age-standardised DALY rate showed a 3.9% reduction to 1760.2 (95%UI: 1671.6–1864.3) in 2015.

**Fig. 2:**
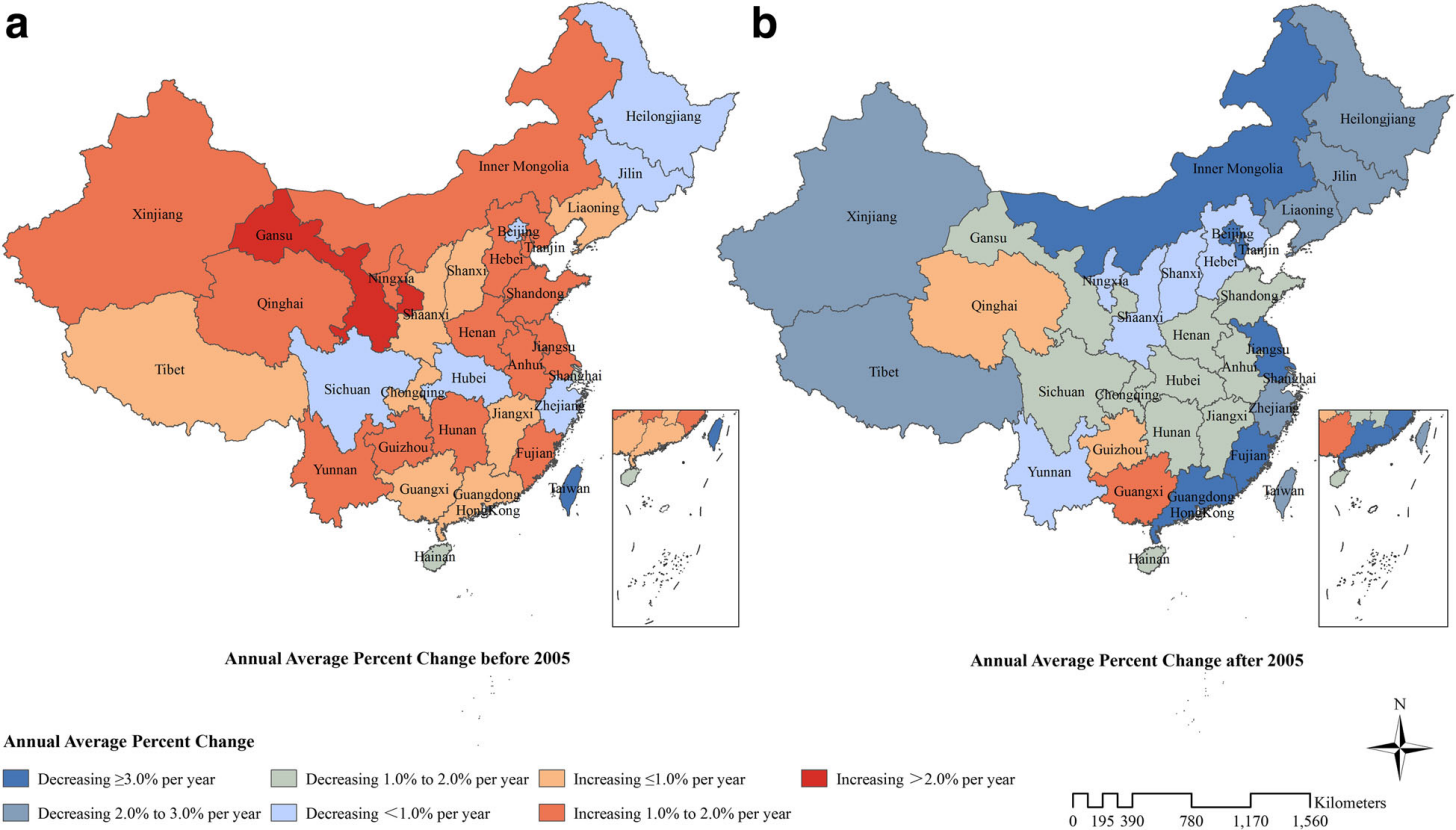
Annual average percent change of age-standardised DALY rates from ischaemic heart disease for both sexes combined in China before 2005 (**a**) and after 2005 (**b**). DALYs = disability-adjusted life years

Table 3 shows the number of DALYs and age-standardised IHD DALY rates for various provinces of China in 1990 and 2015. IHD DALYs for all provinces increased except in Macao. Xinjiang had the highest age-standardised DALY rate at 3040.8 per 100,000 people (95%UI: 2488.8–3735.4) in 2015, followed by Qinghai and Heilongjiang, while Shanghai, Zhejiang and Hong Kong had the lowest rates. Age-standardised DALY rates for southern provinces were lower than northern provinces, especially in southeastern coastal provinces (Fig. 1b). Nineteen provinces showed a downward trend for age-standardised DALY rates with the most rapid decline observed in Macao, Hong Kong, and Beijing. The remaining 14 provinces showed an upward trend, where Qinghai, Guangxi and Yunnan had the fastest growth rate. Before 2005, the age-standardised DALY rates for most provinces increased except in ten provinces including Macao, Hong Kong, Hainan, Shanghai, Sichuan, Zhejiang, Beijing, Hubei, Jilin and Heilongjiang. However, after 2005, the age-standardised DALY rates for the majority of provinces declined except in Guangxi, Qinghai, and Guizhou (Fig. 2and Additional file 1).

**Table 3:** Number of DALYs and age-standardised DALY rates from ischaemic heart disease with percent change, 1990–2015, in provinces of China

Province	Number of DALY (thousand)			Age-standardized DALY rate per 100,000 people		
	1990	2015	% change	1990	2015	% change
China	1374.6 (1294.0–1464.9)	2576.5 (2439.1–2737.4)	87.4	1832.4 (1728.7–1948.7)	1760.2 (1671.6–1864.3)	−3.9
Anhui	520.8 (411.4–661.6)	1057.5 (878.0–1264.8)	103.0	1497.6 (1207.2–1870.7)	1592.9 (1331.6–1895.2)	6.4
Beijing	159.0 (126.7–201.3)	266.1 (224.9–320.0)	67.3	1939.7 (1566.7–2398.3)	1151.9 (982.1–1369.1)	− 40.6
Chongqing	143.8 (113.5–181.0)	462.3 (356.0–581.1)	221.5	1280.5 (1027.7–1588.0)	1294.9 (1002.3–1615.8)	1.1
Fujian	244.5 (195.9–308.2)	362.5 (292.0–435.5)	48.3	1376.1 (1116.7–1710.6)	1009.8 (818.3–1206.1)	−26.6
Gansu	205.5 (160.8–252.1)	466.1 (375.0–567.3)	126.8	1630.4 (1309.0–1969.3)	1835.6 (1494.7–2194.7)	12.6
Guangdong	639.6 (501.8–797.2)	1261.2 (1076.4–1505.2)	97.2	1685.2 (1334.3–2088.1)	1364.1 (1174.2–1600.6)	−19.1
Guangxi	463.9 (368.3–588.2)	1088.1 (885.8–1329.0)	134.5	1787.8 (1435.2–2241.3)	2236.0 (1826.4–2717.6)	25.1
Guizhou	286.4 (226.2–365.5)	667.0 (521.9–858.0)	132.9	1572.3 (1259.6–1972.2)	1922.0 (1513.8–2449.3)	22.3
Hainan	79.8 (63.3–101.6)	123.2 (94.8–159.8)	54.3	2025.2 (1624.4–2519.4)	1398.5 (1085.1–1796.2)	−30.9
Hebei	888.2 (681.8–1137.1)	1822.5 (1485.1–2261.9)	105.2	2138.2 (1665.4–2702.3)	2345.2 (1931.9–2865.0)	9.7
Heilongjiang	737.4 (559.1–930.4)	1270.2 (1045.4–1525.8)	72.2	3686.8 (2866.3–4566.4)	2859.9 (2395.7–3390.3)	−22.4
Henan	1269.1 (1006.0–1574.7)	2242.4 (1832.3–2751.4)	76.7	2249.4 (1800.7–2763.2)	2417.6 (1998.5–2943.1)	7.5
Hong Kong Special Administrative Region of China	69.6 (65.5–76.3)	90.8 (81.5–102.2)	30.6	1369.7 (1290.2–1505.6)	786.0 (705.7–881.4)	−42.6
Hubei	642.7 (514.3–805.1)	1034.8 (853.4–1248.9)	61.0	1909.7 (1551.5–2345.8)	1662.0 (1381.1–1967.2)	−13.0
Hunan	706.9 (554.5–878.8)	1549.0 (1284.5–1859.2)	119.1	1798.3 (1426.3–2218.2)	2084.4 (1741.6–2484.0)	15.9
Inner Mongolia	352.0 (277.0–451.1)	648.1 (526.2–790.8)	84.1	2908.1 (2332.2–3658.3)	2467.7 (2026.8–2961.2)	− 15.1
Jiangsu	489.5 (384.3–618.0)	843.0 (710.1–994.9)	72.2	1000.0 (791.4–1250.3)	875.3 (739.5–1028.2)	− 12.5
Jiangxi	378.3 (299.1–477.3)	656.8 (535.5–790.4)	73.6	1771.3 (1411.3–2196.9)	1564.9 (1289.6–1858.2)	−11.7
Jilin	538.8 (419.2–683.0)	854.0 (667.2–1114.2)	58.5	3666.7 (2915.4–4577.5)	2752.4 (2188.9–3502.6)	−24.9
Liaoning	637.8 (499.8–798.2)	1117.1 (943.8–1341.7)	75.1	2321.8 (1845.0–2863.8)	1979.6 (1681.8–2345.0)	−14.7
Macao Special Administrative Region of China	6.7 (5.9–7.5)	6.6 (5.3–8.3)	−1.1	2679.6 (2355.6–2979.1)	1098.2 (870.6–1364.4)	−59.0
Ningxia	48.6 (36.9–60.3)	148.4 (113.0–191.0)	205.3	2163.5 (1695.5–2641.8)	2649.4 (2069.2–3343.4)	22.5
Qinghai	48.2 (37.1–62.3)	138.9 (103.1–182.5)	187.9	2156.2 (1703.9–2737.8)	2872.5 (2208.7–3675.4)	33.2
Shaanxi	418.8 (332.2–525.8)	899.5 (663.0–1142.5)	114.8	2065.8 (1669.0–2559.3)	2306.1 (1737.2–2879.4)	11.6
Shandong	1114.0 (909.5–1368.5)	2400.2 (1984.6–2879.7)	115.5	1885.6 (1553.7–2303.5)	2138.7 (1787.0–2548.1)	13.4
Shanghai	108.1 (87.3–131.2)	161.0 (132.1–196.5)	49.0	881.2 (715.2–1062.1)	524.4 (434.7–638.4)	− 40.5
Shanxi	404.4 (316.4–508.7)	809.3 (600.2–1047.5)	100.1	2168.0 (1724.6–2701.7)	2209.8 (1678.3–2811.7)	1.9
Sichuan	1057.8 (836.1–1326.3)	1117.3 (874.9–1389.6)	5.6	1529.5 (1244.9–1902.9)	1167.6 (923.1–1444.6)	−23.7
Tianjin	155.1 (122.5–194.6)	285.8 (236.6–343.0)	84.3	2296.6 (1845.5–2840.7)	1912.2 (1605.1–2266.2)	−16.7
Tibet	35.3 (26.9–47.0)	50.2 (40.1–63.9)	42.4	2739.3 (2113.4–3645.6)	2328.8 (1890.5–2913.8)	−15.0
Xinjiang	270.9 (210.0–348.0)	602.3 (481.9–754.3)	122.4	3245.9 (2557.4–4111.5)	3040.8 (2488.8–3735.4)	−6.3
Yunnan	346.7 (274.6–439.9)	856.7 (696.1–1062.4)	147.1	1589.4 (1273.6–1993.5)	1962.7 (1614.1–2407.5)	23.5
Zhejiang	278.2 (222.8–342.9)	406.6 (334.2–489.4)	46.2	932.8 (753.9–1133.3)	635.9 (524.2–763.4)	− 31.8

DALYs = disability-adjusted life years

### Risk factors

**Fig. 3:**
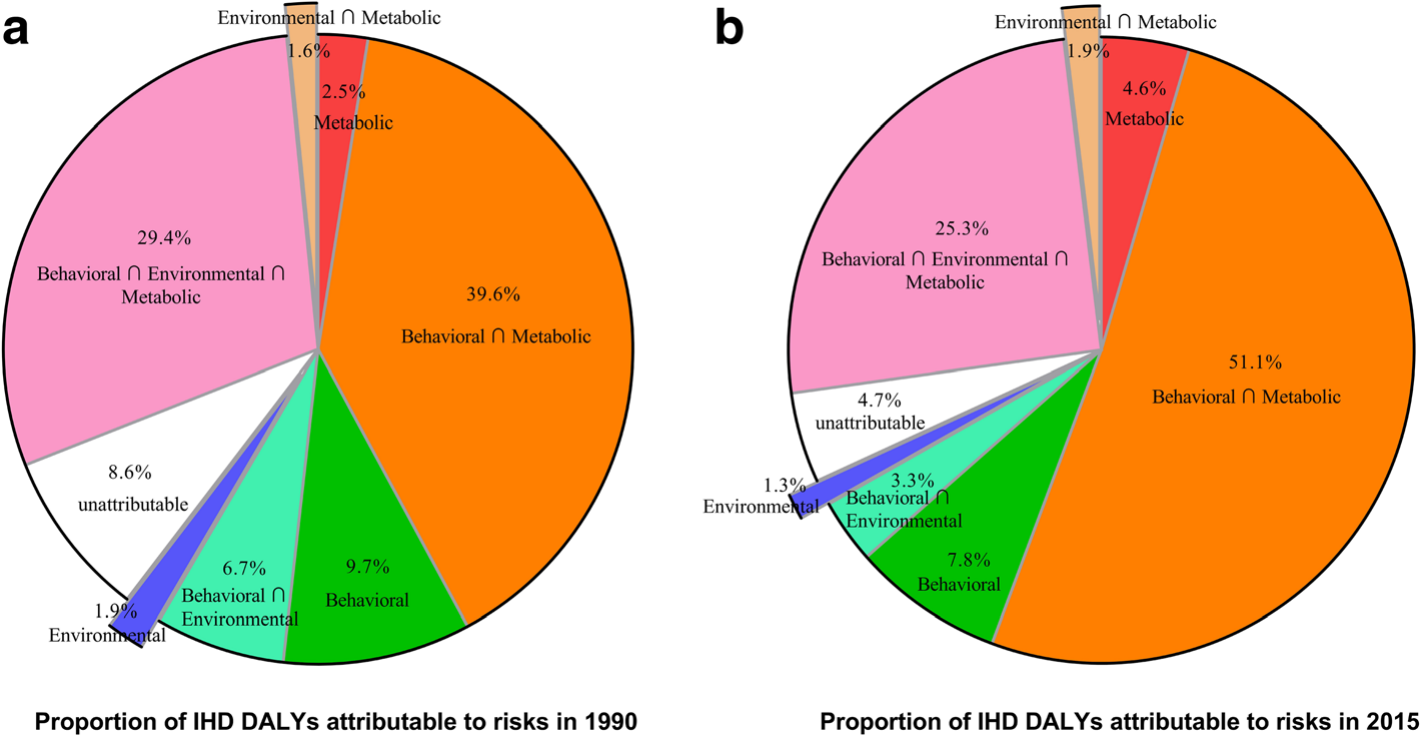
Proportion of DALYs from ischaemic heart disease attributable to metabolic, environmental and behavioural risk factors and their overlaps, for both sexes combined during 1990 to 2015. DALYs = disability-adjusted life years

In China, 95.3% of IHD DALYs (24,561.9 thousand, 95%UI: 23,193.9–26,122.8) were attributable to metabolic, environmental, and behavioural risks and their overlaps in 2015 compared to 91.4% of IHD DALYs (12,560.7 thousand, 95%UI: 11,813.6–13,405.6) in 1990. From 1990 to 2015, the proportion of the three metabolic-related risks and overlaps increased while other clusters decreased, including metabolic risks alone, interaction of behavioural and metabolic risks, and interaction of metabolic and environmental risks (Fig. 3). A substantial portion of IHD DALYs was attributable to behavioural-related risks, including behavioural risks alone (7.8%), interaction of behavioural and metabolic risks (51.1%), and interaction of behavioural and metabolic and environmental risks (25.3%).

**Fig. 4:**
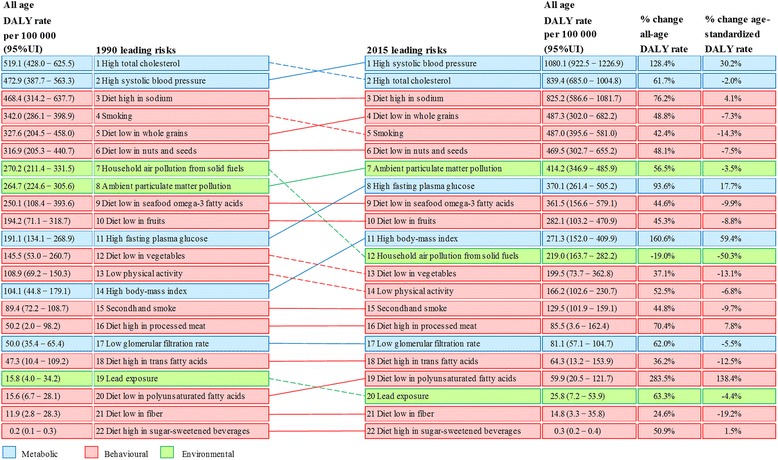
The 22 leading risk factors for DALYs from ischaemic heart disease for both sexes combined in 1990 and 2015 in China. DALYs = disability-adjusted life years

Figure 4 shows the changes to the 22 leading risk factors for IHD DALYs between 1990 and 2015 in China. In 2015, the five leading IHD risk factors and their DALY rates per 100,000 people were high systolic blood pressure (1080.1, 95%UI: 922.5–1226.9), high total cholesterol (839.4, 95%UI: 685.0–1004.8), diet high in sodium (825.2, 95%UI: 586.6–1081.7), diet low in whole grain (487.3, 95%UI: 302.0–682.2), and smoking (487.0, 95%UI: 395.6–581.0). DALY rates for all risk factors increased between 1990 and 2015 except for household air pollution from solid fuels. The largest increase in the DALY rate occurred for diet low in polyunsaturated fatty acids (283.5%), followed by high body mass index (160.6%) and high systolic blood pressure (128.4%). However, only seven risk factors increased in age-standardised IHD DALY rates, while the remaining 15 risk factors declined. The age-standardised IHD DALY rate for household air pollution from solid fuels showed the greatest decline (50.3%). Six risk factors, including high systolic blood pressure (2nd to 1st), diet low in whole grains (5th to 4th), ambient particulate matter pollution (8th to 7th), high fasting plasma glucose (11th to 8th), high body-mass index (14th to 11th), and diet low in polyunsaturated fatty acids (20th to 19th), increased in rank between 1990 and 2015.

## Discussion

### Main findings

In this study, we found that the number of deaths and DALYs from IHD have increased significantly in China since 1990, but age-adjusted DALY rate has declined. The IHD burden increased in most provinces before 2005 but declined after 2005. Geographically, the IHD burden in southern provinces were lower than northern provinces, especially in southeastern coastal provinces. Furthermore, 95.3% of the IHD burden in China was attributable to environmental, behavioural and metabolic risk factors. The top five IHD risks were high systolic blood pressure, high total cholesterol, diet high in sodium, diet low in whole grains, and smoking.

### Mortality and disability-adjusted life years

IHD was the second leading cause of disease burden in China only after stroke. IHD deaths and DALYs have greatly increased for both sexes since 1990. Moreover, China made the second greatest contribution to global IHD burden after India, accounting for 15.7% of total global IHD DALYs in 2015 [5]. These findings call for comprehensive approaches to prevent and reduce the IHD burden in China.

The IHD death rate and the DALY rate increased greatly, but the age-adjusted death rate and DALY rate showed no obvious increases between 1990 and 2015. These findings were mainly caused by population growth and ageing during this period. YLLs were the main component of IHD burden in China with over 90% of DALYs attributed to YLLs. A global level assessment has shown that YLL rates for CVDs increased with the Socio-demographic Index for countries and regions in the middle of the socio-demographic rankings [4]. Most provinces in China remain in the middle of the socio-demographic rankings despite experiencing an increase in the Socio-demographic Index since 1990 [4]. Evidence has also shown that IHD patients in low- and middle-income countries have less access to affordable and high quality CVD medications [22]. All of these findings imply that, in most provinces of China, economic growth and social development have led to an increase in the life expectancy to allow individuals to survive long enough to develop IHD; however, many of them are not able to access to optimal medical or surgical treatments for IHD. Therefore, both the YLL rate and YLD rate from IHD in China have increased significantly since 1990. However, age-standardised YLL and DALY rates have declined, implying improvements in medical care and social security in China that provided more available and affordable treatments for IHD patients.

Differences in the burden of IHD existed between sexes, time periods, and provinces in China. With respect to gender, the IHD burden was higher and increased more rapidly among males than females. Because females are more sensitive to heath information, females are more likely to seek healthcare and have better access to primary prevention compared to males [23]. Regarding time period, the IHD burden in most provinces increased before 2005 but declined afterwards. This transition occurred for multiple reasons. The increase before 2005 was mainly caused by the increase in prevalence of IHD risk factors such as hypertension, dyslipidaemia, and ambient particulate matter pollution [15]. The IHD burden declined after 2005 partly due to the implementation of many important strategies related to tobacco use, diet, physical activities, and harmful use of alcohol to prevent and control NCDs [24–26]. In addition, improvement in IHD treatments and the application of cardiac rehabilitation in recent years have led to great advances in reducing the mortality in IHD patients. Regarding the various provinces, the IHD burden was lower among southern provinces than northern provinces, especially in southeastern coastal provinces. These regional disparities are observed partly due to the differences in exposure to metabolic and behavioural risk factors, environmental qualities, meteorological conditions, and household income levels between different regions. Southeastern coastal provinces experienced a lighter IHD burden, as these provinces have been shown to have lower prevalence of hypertension and high total cholesterol [27–29], a lower smoking rate [30], lower air pollution [31], higher household income [32], warm climate conditions and eating habits similar to the Mediterranean diet or Japanese diet [33].

### Risk factors

In 2015, over 90% of IHD DALYs were attributable to risk factors that are controllable through reducing and managing related metabolic, behavioural and environmental risk factors. Behavioural risk factors remained the main source of IHD burden in China, but proportion of DALYs attributable to metabolic risk factors has increased previously since 1990, which should be brought to the forefront.

Over the past 30 years, the economy and society in China have undergone rapid improvement with demographic transitions and lifestyle changes with a large impact on population health. Exposure to metabolic and behavioural risk factors has increased to high levels in recent years. A recent report on CVDs in China showed that 25.2% of Chinese adults (approximately 270 million) suffered from hypertension and 33.6% had high-normal blood pressure, but their knowledge rate, treatment rate and control rate for hypertension were only 46.5%, 41.1%, and 13.8%, respectively [33]. Approximately 10% of Chinese adults had high total cholesterol and 25% had high triacylglycerol. The smoking rate was 27.7% in resident aged 15 years and 52.1% in males. A 14.5 g daily average salt intake in Chinese adults was much higher than 5 g recommended by WHO [34]. Moreover, approximately 9.7% of adults had diabetes, and the overweight and obesity rates for adults were 30% and 11.9%, respectively [33]. These findings imply the immense potential of prevention through comprehensive risk factor modification to reduce the IHD burden in China [35, 36].

### Study limitations

Our study has several limitations. First, since data for this study were mainly obtained from the GBD 2015 study, all limitations of the GBD 2015 methods outlined elsewhere also apply here [4–7]. The GBD 2015 study used big data analytic technologies to integrate heterogeneous data and estimate burden of diseases and risk factors. Although many methods and processes were used to reduce bias, including misclassification corrections, redistribution of garbage codes, and noise reduction, it was difficult to thoroughly avoid inaccuracy. Therefore, our results on the IHD burden in provinces of China should be interpreted carefully, as the data we used were estimates. Second, accurate data such as follow-up data on non-fatal IHD outcomes and prevalence of IHD risk factors by provinces are very limited. Due to the insufficiency of provincial data regarding risk factors, our study lacked analysis of regional differences in IHD risk factors, which should be conducted in the future.

### Implication for policies

Our findings indicate priorities for comprehensive approaches to reduce the IHD burden in China. First, policies should be created to reduce population exposure to major IHD risk factors such as hypertension preventing and curing, cholesterol lowering, advocating healthy diets such as the Mediterranean diet, smoking cessation, controlling ambient particulate matter pollution, etc. Second, it is essential to monitor and manage individuals at high risk for IHD such as those with high blood pressure, dyslipidaemia, high body mass index and hyperglycaemia, especially in males and the elderly, on the community level. Furthermore, improvement in medical care, IHD treatments and social security will help provide more available, affordable, and high quality treatments for IHD patients. Lastly, health education programmes need to be launched to increase awareness of the importance of controlling IHD and personal preventive strategies among the general population.

## Conclusion

Although age-standardised DALY rate from IHD declined from 1990 to 2015 in China, population growth and ageing have led to sharp increases in deaths and DALYs from IHD. Regional disparities in IHD burden were observed among provinces of China. The IHD burden in most southern provinces was lower than that of northern provinces, especially in southeastern coastal provinces. Metabolic, behavioural, and environmental risk factors contributed to the majority of the IHD burden. The characteristics of IHD burden and IHD risk factors provide guidance for decision makers to formulate targeted preventive policies and interventions.

## Additional file

Additional file 1: ᅟ. (DOCX 18 kb)

## Abbreviations

95%UI: 95% uncertainty interval
CVDs: Cardiovascular diseases
DALYs: Disability-adjusted life years
GBD 2015: Global burden of disease, injuries, and risk factors 2015 study
IHD: Ischaemic heart disease
NCDs: Non-communicable diseases.
YLDs: Years lived with disability
YLLs: Years of life lost
