# Supplementary material for: Burden of Ischaemic heart disease and attributable risk factors in China from 1990 to 2015: findings from the global burden of disease 2015 study
Source: BMC Cardiovasc Disord. 2018 Feb 2;18:18. doi: 10.1186/s12872-018-0761-0 (PMC6389214; doi:10.1186/s12872-018-0761-0)
Supplement: Supplementary file 1 — ᅟ. (DOCX 18 kb) [file 12872_2018_761_MOESM1_ESM.docx]

Annual average percent change for age-standardised DALY rate from ischaemic heart disease in provinces of China before 2005 and after 2005 was displayed in the table as below.

| Location | Annual average percent change (%) | |
| --- | --- | --- |
|  | Before 2005 | After 2005 |
| Anhui | 1.3 | -1.3 |
| Beijing | -0.8 | -3.9 |
| Chongqing | 0.9 | -1.3 |
| Fujian | 1.1 | -4.7 |
| Gansu | 2.0 | -1.8 |
| Guangdong | 0.7 | -3.1 |
| Guangxi | 0.7 | 1.3 |
| Guizhou | 1.3 | 0.1 |
| Hainan | -1.3 | -1.8 |
| Hebei | 1.3 | -0.9 |
| Heilongjiang | -0.1 | -2.4 |
| Henan | 1.8 | -1.9 |
| Hong Kong Special Administrative Region of China | -3.2 | -0.7 |
| Hubei | -0.1 | -1.2 |
| Hunan | 1.7 | -1.1 |
| Inner Mongolia | 1.2 | -3.4 |
| Jiangsu | 1.2 | -3.0 |
| Jiangxi | 0.1 | -1.3 |
| Jilin | -0.3 | -2.4 |
| Liaoning | 0.6 | -2.4 |
| Macao Special Administrative Region of China | -3.5 | -3.5 |
| Ningxia | 1.8 | -0.7 |
| Qinghai | 1.7 | 0.4 |
| Shaanxi | 0.8 | -0.1 |
| Shandong | 1.5 | -1.0 |
| Shanghai | -1.5 | -2.8 |
| Shanxi | 0.4 | -0.4 |
| Sichuan | -0.9 | -1.3 |
| Tianjin | 1.5 | -3.9 |
| Tibet | 0.4 | -2.2 |
| Xinjiang | 1.3 | -2.5 |
| Yunnan | 1.6 | -0.2 |
| Zhejiang | -0.7 | -2.7 |

The annual average percent change was calculated by formulae as below:

Where, all the rate were present to the age standardised DALY rate for provinces of China in 1990, 2005, and 2015.
